# How the Russian Red Cross Gets Its Wants Supplied

**Published:** 1916-12-16

**Authors:** Sonia E. Howe


### HOW THE RUSSIAN RED CROSS GETS ITS WANTS SUPPLIED.

By Mrs. Sonia E. Howe.

Those Government Departments which in England are designated by the name of Foreign Office, Home Office, and Board of Trade are called in Russia "Ministries," and someone suggested, not inaptly, that the Red Cross could be described as the Ministry of Mercy.

It was my privilege to be personally conducted over the Central Depôt of the Red Cross in Petrograd, and what I saw there filled me with amazement. In time of peace the Russian Red Cross finds an outlet for its activity in sending relief to famine-stricken districts, or in fighting epidemics in the outlying districts of the vast Empire. My guide, Professor O. v. Petersen, Vice-President of the Council of the depôt, told me that many lessons were learnt by the Committee of the Red Cross during the Japanese War, and perhaps the most important one was that, to meet efficiently the demands of war, it is imperative to prepare for them in time of peace. After the conclusion of the Japanese War a few members of the Red Cross Committee, chief among them B. C. Ordin, a former Chamberlain to His Majesty the Emperor, and Professor O. v. Petersen set to work to found the Council of the Central Depôt. Having received permission to do so, they called in all the materials left over from the war, and had them carefully sorted and stored in a block of warehouses, rented at a cost of 3,000 roubles per month. The accumulated stores were gradually sold off to other institutions, and fresh material bought with the money realised by the sale. A complete scheme of preparedness was soon evolved, and model types of field hospitals, with all the necessary appliances worked out in plan, catalogued, and then gradually all the necessaries were actually prepared. The Council soon launched out into a more ambitious enterprise, and a large piece of ground was purchased on the outskirts of Petrograd, and at a cost of 700,000 roubles the wonderful Central Depôt of the Red Cross was built and fitted out. It is a misnomer to call it a depôt; a more accurate name would be a general supply store and manufactory, for

### THE RUSSIAN RED CROSS—(*continued*).

the Central Depôt does more than merely supply the articles : it actually manufactures them on the spot.

To walk through the various workrooms and departments is like inspecting so many factories, laboratories, workshops, warehouses of clothing and underlinen, of hardware and saddlery. I saw close upon 200 neatly clad young girls preparing bandages with the aid of first-class machinery. The bandages were then sterilised, rolled up, and packed as in a factory. In another place one of the *sanitars* engaged at the depôt was working a machine which cut out sixty pairs of calico trousers at one turn of the handle. I walked through storerooms packed up to the very ceiling with blankets, warm vests, and woollen hosiery. "Let the winter come," said my guide; "we do not fear it; we have everything ready." In the saddlery store was every appurtenance for horses and ambulance vans. There are more than eighty-three such ambulance units, each with 180 horses, at present in use at the Front. I passed through a large room in which hardware was stored, principally boilers for drinking-water; and whereas the foreign stills, of which I saw some fine samples, cost £90 each, one, invented by the ingenious manager of the depôt, costs ten guineas only, and does equally good service. It is due to these stills that there is so little illness among the Russian troops, who thus not only drink boiled water, but actually distilled water. The manager showed me with pride field medicine chests which, if any bombs or shells burst too close by, can be folded up at a moment's notice and strapped to the back of a horse to be carried to a safer place. I also saw the surgeon's instrument cabinets, equally portable. In fact, everything is so carefully planned out as to require the minimum space for transport; for example, a thousand field-hospital bedsteads could be packed into one van were it strong enough to bear the weight, and whereas in the Japanese War twelve trucks were required for a given quantity, now five suffice. Efficiency, economy of space and time, is not only aimed at, but secured. Everything is ready to hand in the depôt, and the request for the dispatch of a complete field hospital can be met just as easily and as speedily as an order for so many thousands of tabloids, bottles of glycerine, or packages of bandages. It is not only that the order is received, but it is actually executed the same day as received. The articles, already packed and ready for use, are promptly placed in trucks which stand inside the warehouse and immediately carried along a private railway line, which is connected with the central railway system. Just as expeditious is the reception of the goods which come to the depôt from every part of the Empire, as well as from allied and from neutral countries. Customs dues on foreign goods are collected on the spot, for a special Customs Office has been established at the depôt in order to avoid delay.

### FURTHERING HOME INDUSTRIES.

In a yard I saw large numbers of iron casks—"carbolic acid from England," I was told. In another warehouse were goods from America, bales of cotton from Japan, and so on. From America, too, came the machinery for making tabloids, and it was most fascinating to watch 500 codein tabloids being turned out in a minute. Owing to the impossibility of getting certain drugs as hitherto from abroad, the energetic Council at once set to work to provide for a regular supply of these drugs by the Russian chemical factories. In fact, the Council has directly helped to further home industries, with the result that a number of articles of every kind formerly supplied by Germany, even such fine instruments as clinical thermometers, etc., are now being turned out as successfully in Russia. It has been a revelation to the Russian manufacturers as to what they are capable of producing, for all they supply to the depôt must be of the best quality. The members of the Council have made it a rule never to accept anything which does not pass the test, and have set themselves the arduous task of personally inspecting the goods. The Council is subdivided into commissions, each devoted to its own speciality, and thus every delivery of goods, whether of tapes, drugs, or boilers, is examined and tested, and if found to be below the standard it is rejected. It is owing to this care in the minutiæ of detail that field hospitals and other Red Cross institutions never find themselves at a loss, for whatever they receive can be relied upon for quality.

### A COSTLY TRANSACTION.

What drastic measures are taken if goods prove not to be what they seem, is characteristically illustrated by the following incident. An offer of so many bales of absorbent cotton-wool had been made by Japan and accepted by the Council. These bales were examined on arrival, and a great many were found to contain ordinary cotton-wool, upon which an immediate request was made to Japan to send men out at once in order to go through the immense quantity of bales. After the Japanese had separated the absorbent from the non-absorbent cotton-wool, the latter was paid for at its real value—that is, at half the price of the absorbent—and since then all Japanese goods delivered to the depôt leave nothing to be desired.

The twenty-four energetic members of the Council who so whole-heartedly give their time and thought to this great work are busy men, renowned experts in their different professions, and all give their time as honorary workers to the depôt. For these gentlemen—Court officials, army and medical men, scientists, manufacturers, and merchants—to come twice a week to the depôt to do their work of inspection or examination proves how faithfully and patriotically they carry out their self-imposed duty towards their wounded fellow-countrymen.

The Russian Red Cross is primarily intended to act as a supplementary agency to the Medical Department of the War Office, upon which in Russia the care of the wounded devolves. On what a gigantic scale this supplementary work is carried out can be judged from the following facts. Thus at the present time the Central Depôt of the Red Cross supplies 2,007 institutions of all kinds, either in the war zone or at the base. The Council of the depôt has fitted out since the outbreak of the war two hospital ships, both of which have unfortunately been sunk by the enemy in the Black Sea. It has provided and sent out to the Front ninety portable *x*-ray installations, some of them fixed upon motor-cars; has equipped twenty-six hospital trains; and has established five factories for the making of artificial ice—all this in addition to the numerous field hospitals. How the demand upon the Council has steadily increased is seen from the fact that whereas in 1914 the expenditure by the Council was 7,000,000 roubles, it rose in 1915 to 20,000,000 roubles, and had already reached a like sum in the first half of 1916.

